# Outcomes of Phaco-viscocanalostomy in Primary Open Angle Glaucoma versus Pseudoexfoliation Glaucoma

**DOI:** 10.18502/jovr.v16i4.9746

**Published:** 2021-10-25

**Authors:** Ebrahim Azaripour, Yaser Khakpour, Reza Soltani-Moghadam, Zahra Moravvej, Abdolreza Medghalchi, Hassan Behboudi, Yousef Alizadeh, Soheil Soltanipour, Shila Kianmehr

**Affiliations:** ^1^Eye Research Center, Department of Ophthalmology, Amiralmomenin Hospital, School of Medicine, Guilan University of Medical Sciences, Rasht, Iran; ^2^GI Cancer Screening and Prevention Research Center, Department of Community Medicine, School of Medicine, Guilan University of Medical Sciences, Rasht, Iran

**Keywords:** Primary Open-angle Glaucoma Intraocular Pressure, Phacoviscocanalostomy, Pseudoexfoliation

## Abstract

**Purpose:**

Viscocanalostomy represents an alternative to standard penetrating glaucoma surgery. The aim of this study is to compare the outcomes of combined phacoemulsification and viscocanalostomy in eyes with primary open-angle glaucoma (POAG) versus eyes with pseudoexfoliation glaucoma (PEXG).

**Methods:**

In this prospective non-randomized comparative study, eyes with cataract and POAG or PEXG were enrolled. Pre- and postoperative data including best corrected visual acuity (BCVA), intraocular pressure (IOP), and the number of antiglaucoma medications administered were recorded at each visit. All patients underwent phacoviscocanalostomy. Complete success was defined as the IOP of 21 mmHg or less without the administration of medication while a qualified success reported the same IOP parameters either with or without the administration of medication.

**Results:**

Fifty-four eyes with POAG and fifty-four with PEXG underwent phacoviscocanalostomy. The mean follow-up time was 23.36 ± 8.8 months (range, 6–40 months). The mean postoperative IOP reduced significantly in both groups, although the mean IOP reduction was significantly greater in PEXG eyes (14.7 ± 8.9 vs 10.1 ± 7.7 mmHg) (*P* = 0.05). At the final follow-up visit, the mean postoperative IOP was 14.1 ± 2.1 and 16.6 ± 3.5 mmHg in the PEXG and POAG eyes, respectively (*P* = 0.001). A complete success rate of 88.9% and 75.9% was achieved in PEXG and POAG eyes, respectively (*P* = 0.07). The qualified success rate was 100% in the PEXG and 85.2% in POAG groups (*P* = 0.03).

**Conclusion:**

Phacoviscocanalostomy achieved significant IOP reduction and visual improvement in both POAG and PEXG patients. Our results indicated that in terms of IOP reduction, this procedure was more effective in treating PEXG.

##  INTRODUCTION

Primary open-angle glaucoma (POAG) and pseudoexfoliation glaucoma (PEXG) are chronic and progressive processes causing optic neuropathy. Pseudoexfoliation syndrome is featured by the deposition of specific fibrillar material in the anterior segment of the eye.^[1,2]^ This sedimentation of material on the trabecular meshwork (TM) may cause glaucoma and subsequent optic neuropathy.^[3]^

Various surgical procedures exist for managing glaucoma; these include penetrating and nonpenetrating filtering procedures and tube shunt surgery.^[4,5]^ Nonpenetrating procedures, such as deep sclerectomy and viscocanalostomy (VCS) have been designed to alleviate the complications of penetrating surgery^[6]^. VCS, as defined by Stegmann et al, involves the injection of high-viscosity sodium hyaluronate into the Schlemm's canal until aqueous outflow drainage is improved.^[7]^ It has been suggested that physiologic aqueous humor drainage may be attained without bleb formation.

Although trabeculectomy has proven to be more effective than nonpenetrating procedures in terms of IOP reduction, it is more prone to complications.^[9]^ VCS releases less inflammatory mediators causing lower rates of bleb failure. Phacoviscocanalostomy represents an alternative to standard phacotrabeculectomy with anti-metabolites.^[8]^ Recent reports show good mid-term results of phacoviscocanalostomy in eyes with medically uncontrolled glaucoma.^[10,11,12]^ In this study, we aim to compare the clinical outcomes of phacoviscocanalostomy with intraocular lens implantation in PEXG and POAG eyes.

##  METHODS

This prospective comparative study was conducted on 108 of 108 eyes with PEXG (54 eyes) and POAG (54 eyes). The criteria used in choosing the participants for the study included eyes with medically uncontrolled POAG and PEXG (IOP > 21 mmHg with maximum medical therapy and glaucomatous visual field defects) and visually significant cataract (visual acuity that significantly affects the patient's daily activities). Patients with other ocular pathologies and history of any ocular surgery or laser procedures were excluded from the study.

Demographic and preoperative data which included best corrected visual acuity (BCVA) in LogMAR, IOP (Goldmann tonometry), gonioscopic (four-mirror glass goniolens), and funduscopic findings were recorded. Standard automated perimetry (Humphrey Field Analyzer, program 24-2) and glaucoma severity staging done according to Hodapp-Parrish-Anderson criteria was also obtained from all patients. Postoperative follow-ups were conducted initially on day seven and subsequently at 1, 3, 6, 12, 24, 30, and 40-month intervals. Complete slit-lamp examinations were performed in each follow-up visit along with BCVA and IOP measurements.

The study was conducted in accordance with the principles of the Declaration of Helsinki and was approved by the local Ethics Committee. Patients were informed about the aim of the study and written informed consent was obtained from all participants before the surgery. At the ophthalmology clinic, all eyes underwent surgery and follow-up examinations, both of which were performed by a single surgeon (EA).

Statistical analysis was calculated using the SPSS V19.1 application. Preoperative and postoperative data were compared using paired sample *t*-test and Wilcoxon sign test (for nonparametric data). Comparison between the two groups was performed using student's *t*-test and the Mann–Whitney *U *test (for nonparametric data). Comparisons of the outcome of the glaucoma at different stages were done using the Kruskal–Wallis test. The frequency of intraoperative and postoperative complications was assessed in both groups. *P*-value < 0.05 was considered to be statistically significant. According to international consensus statements, complete success was characterized as an IOP ≤ 21 mmHg without antiglaucoma medication while qualified success was determined as achieving the same IOP values but either with or without antiglaucoma medication. Failure was described as an IOP > 21 mmHg with the administration of medication, or when an eye required further glaucoma drainage surgery.^[13]^

### Surgical Technique

Surgical procedures were performed either utilizing peribulbar or general anesthesia depending on the patient's medical history. Prior to performing VCS, patients underwent standard phacoemulsification with intraocular lens implantation. Phacoemulsification was performed through a temporal 2.8 mm clear cornea incision. After executing continuous circular capsulorhexis (CCC), the nucleus was emulsified using “stop and chop” or “divide and conquer” techniques. After complete nuclear and cortical material removal, a foldable intraocular lens was inserted into the capsular bag. In order to perform VCS, a fornix-based conjunctival peritomy was made and a square-shaped superficial scleral flap (5.0 х 5.0 mm) was created by a crescent blade. A second deeper scleral flap (4.0 х 4.0 mm) was dissected and continued anteriorly to the scleral spur to expose the trabeculo-descemet membrane (TDM). The deep scleral flap and the roof of Schlemm's canal were cut to facilitate aqueous humor drainage. Sodium hyaluronate 1.4% (Healon GV) was then injected into the Schlemm's canal. The superficial scleral flap was closed tightly with two interrupted 10-0 nylon sutures, and the conjunctival wound was sutured with 10-0 nylon.

##  RESULTS

A total of 108 eyes of 108 patients with PEXG (54 eyes) and POAG (54 eyes) were included in the study. The mean age of the participants was 70.3 ± 8.03 years and 61 (56.5%) of them were male. All patients completed postoperative follow-up for at least 12 months. The mean follow-up time after surgery was 23.36 ± 8.8 months (range 12–40 months). The demographic and baseline data of the two groups is shown in Table 1. There was no significant difference between the PEXG and the POAG groups in terms of glaucoma severity (*P* = 0.585). The gonioscopy results revealed slightly narrower angles in the PEXG group as compared to the POAG group, although this difference was not statistically significant (79.6% in PEXG vs 100% in POAG were classified as open-angle, *P =* 0.057).

**Table 1:** Demographic data, baseline, and postoperative data at final follow-up visit in the study groups


	**PEXG ** ***(n=54)***	**POAG ** ***(n=54)***	***P*** **-value**
**Age (yr)**	72.0 ± 7.8	68.5 ± 7.9	0.02
**Gender (male/female)**	33/21	28/26	0.33
**Preoperative BCVA (logMAR)**	0.97 ± 0.4	0.91 ± 0.4	0.32*
**Postoperative BCVA (LogMAR)**	0.38 ± 0.35	0.39 ± 0.35	0.88*
**Preoperative IOP (mmHg)**	28.5 ± 9.0	24.0 ± 8.7	0.004*
**Preoperative antiglaucoma medication (n)**	1.06 ± 1.02	1.5 ± 1.32	0.07
**Postoperative antiglaucoma medication (n)**	0.11 ± 0.31	0.28 ± 0.57	0.06
**Follow-up time (months)**	23.7 ± 8.2	22.9 ± 9.4	0.67
**Cup-to-disc ratio**	0.84 ± 0.1	0.85 ± 0.1	0.85
**Severity, ** ***n*** ** (%)**		0.585 ${}^{†}$
Mild	4 (7.4)	6 (11.1)	
Moderate	15 (27.8)	11 (20.4)	
Severe	35 (64.8)	37 (68.5)	
Data is expressed as Mean ± SD POAG, primary open-angle glaucoma; PEXG, pseudoexfoliation glaucoma; BCVA, best-corrected visual acuity; IOP, intraocular pressure; SD, standard deviation ${}^{*}$ Mann–Whitney *U* test; ${}^{†}$ Chi-square test			

**Table 2:** Mean IOP (mmHg) over 40 months (ms) follow-up in the two study groups


	**1 week ** *n =108*	**1 m ** *n =108*	**3 ms ** *n =108*	**6 ms ** *n =108*	**12 ms ** *n =108*	**24 ms** *n =92*	**30 ms ** *n =84*	**40 ms ** *n =76*
**PEXG**	10.5 ± 3.9	13.0 ± 4.3	13.7 ± 2.7	16.4 ± 2.3	14.1 ± 2.3	14.5 ± 2.1	15.2 ± 2.1	13.5 ± 1.9
**POAG**	9.9 ± 3.1	12.9 ± 3.0	13.2 ± 2.5	15.4 ± 4.6	15.5 ± 3.0	16.8 ± 3.7	16.3 ± 2.8	15.8 ± 1.3
***P*** -value*	0.35	0.33	0.45	0.62	0.009	0.001	0.17	0.08
Data is expressed as Mean ± SD POAG, primary open-angle glaucoma; PEXG, pseudoexfoliation glaucoma *Students *t*-test								

**Table 3:** Intraoperative and postoperative complications


**Complications**	**PEXG ** ***n*** ** (%)**	**POAG ** ***n*** ** (%)**
Descemet membrane microperforation	3 (5.6)	2 (3.7)
Fibrin reaction	5 (9.2)	3 (5.6)
Zonular dehiscence	3 (5.6)	0
POAG, primary open-angle glaucoma; PEXG, pseudoexfoliation glaucoma		

All patients had a significant improvement in BCVA, postoperatively (*P* = 0.001). There was no statistically significant difference in terms of postoperative BCVA between the POAG and the PEXG groups (*P* = 0.88).

**Figure 1:**
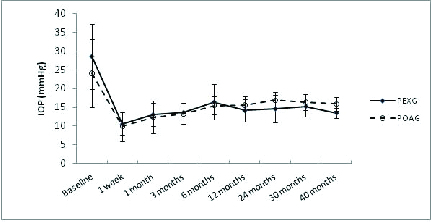
Mean intraocular pressure (IOP) over time in two groups. PEXG, pseudoexfoliation glaucoma; POAG, primary open angle glaucoma.

The mean IOP decreased significantly one week after surgery in both groups (*P* = 0.001) and remained significantly lower than its preoperative value at all follow-up visits [Figure 1]. The mean IOP was significantly lower in the PEXG group as compared to the POAG group at the 12- and 24-month follow-up visits. This trend continued until the last follow-up visit although not statistically significant [Table 2]. At the final follow-up, the mean postoperative IOP reduction in the PEXG group was significantly greater than the POAG group (14.7 ± 8.9 vs 10.1 ± 7.7 mmHg, *P* = 0.01).

**Figure 2:**
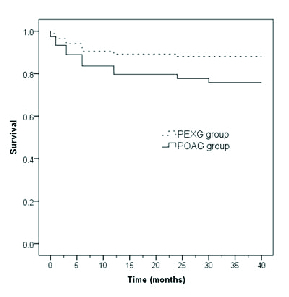
Kaplan–Meier survival curve of complete success (IOP < 21 mmHg without medication) in pseudoexfoliation glaucoma (PEXG) and primary open-angle glaucoma (POAG).

Overall, the number of antiglaucoma medications required reduced significantly after surgery (1.2 ± 1.2 to 0.12 ± 0.46, *P* = 0.001), although not statistically different between the two study groups [Table 1].

The most common intraoperative and postoperative complication of phacoviscocanalostomy was microperforation of the TDM and fibrin formation, respectively [Table 3]. At the final follow-up, complete success was noted in 88.9% (48 eyes) and 75.9% (41 eyes) of the PEXG and POAG patients, respectively (*P* = 0.07). Qualified success was achieved in all eyes (100%) of the PEXG group and 85.2% of the POAG group (*P* = 0.03). In both the PEXG and POAG groups, complete success rates were not significantly different among the varying stages of glaucoma (*P =* 0.587 for stages of PEXG and *P = *0.252 for stages of POAG).

The cumulative probability estimated using the Kaplan–Meier survival curve of complete success (IOP < 21 mmHg without medication) in the study groups is illustrated in Figure 2. A moderately stable level of surgical success was achieved as was noted during the follow-up periods. No patients required additional glaucoma surgery.

##  DISCUSSION

Viscocanalostomy in comparison to trabeculectomy, is a nonpenetrating technique known to decrease intra- and postoperative complications of glaucoma surgery.^[14,15,16]^ Owing to its pathophysiology, PEXG eyes are more prone to trabeculectomy-related complications.^[17,18]^ The blood–ocular barrier dysfunction and iris stromal vasculopathy may contribute to postoperative inflammation, fibrin formation, and IOP elevation.^[19]^

Stegmann et al in 1999 reported the first study of VCS on 214 eyes with open-angle glaucoma (OAG) with a mean follow-up of 35 months.^[7]^ They achieved a complete success rate (IOP ≤ 22mmHg without medication) and a qualified success rate (IOP ≤ 22 mmHg with medication) of 82.7% and 89%, respectively. Another study in 2004 reported a complete success rate of 35.3% and a qualified success rate of 73.9% after three years, following the VCS in POAG eyes.^[20]^ Performing VCS without phacoemulsification, in addition to the reported higher preoperative IOP (36.0 ± 8.0 mmHg) as compared to our study (24.0 ± 8.7 mmHg) may be reasons for the lower success rates achieved in their study. The combined procedures of phacoemulsification and nonpenetrating glaucoma surgery is being used frequently as it has produced good evidence of visual improvements and long-term IOP control.^[16,21]^ A 12-year follow-up study by Gunenc et al showed superior success rates in eyes undergoing phacoviscocanalostomy as compared with performing VCS on its own.^[22]^

In our present study, we noted significant reduction in the mean IOP levels after performing phacoviscocanalostomy in both POAG and PEXG eyes. However, significantly lower IOP levels were achieved in PEXG eyes at the 12- and 24-month follow-up visits. This finding may be partly due to significantly higher preoperative IOP in the PEXG patients. At all postoperative follow-ups, a similar comparative study reported significantly lower mean IOP in the PEXG group after performing phacoviscocanalostomy.^[23]^

In a retrospective one-year study, Moghimi et al reported a 37% complete success rate (defined as IOP ≤ 21 mmHg without medication) in 46 OAG eyes (including PEXG) having undergone phacoviscocanalostomy.^[24]^ In another study, eyes with advanced glaucoma having undergone phacoviscocanalostomy achieved complete and qualified success rates of 30.6% and 80%, respectively.^[25]^ Stangos et al performed phacoviscocanalostomy on 50 eyes with medically uncontrolled OAG and clinically significant age-related cataract. The results reported an overall success rate (IOP ≤ 20 mmHg with or without medication) of 82% and a complete success rate (IOP ≤ 20 mm Hg without medication) of 67% at the 36-month follow-up visit.^[26]^ In comparison to the aforementioned studies,^[24,25,26]^ we experienced higher rates of complete and qualified success. Our study also indicated higher rates of complete success in the PEXG as compared to that of the POAG eyes (88.9 vs 75.9%). All PEXG eyes in our study and 85.2% of the POAG eyes achieved qualified success. Likewise, Awadalla et al reported complete surgical success of 93.3% in PEXG eyes and of 83.3% in POAG eyes.^[23]^ Their study resulted in qualified success of all patients in both groups. Wishart et al also achieved higher complete success rates (defined as IOP ≤ 18 mmHg without medication) in PEXG as compared to POAG eyes after a mean follow-up of six years (95% and 76%, respectively), they also experienced 100% qualified success in PEXG and 90.2% in POAG eyes.^[27]^ According to our current study and the studies mentioned above, phacoviscocanalostomy success rates were higher in PEXG as compared to POAG patients.^[13,14,23]^

The type of glaucoma may also influence IOP reduction after cataract surgery. Numerous studies over the past few decades have shown that cataract surgery leads to a sustained decrease in IOP in POAG and pseudoexfoliation patients.^[28]^ Masis et al performed a systematic review and meta-analysis of the clinical data to estimate the net effect of cataract surgery on IOP. A total of 37 treatment arms from 32 different studies from January 1997 to January 2017 were included. For angle-closure glaucoma, results showed an IOP decrease of - 6.4 mmHg (95% CI: - 9.4 to - 3.4) at final follow-up (12 months and longer). For the OAG group, there was an overall IOP change of - 2.7 mmHg (95% CI: - 3.7 to - 1.7) from the baseline and for PEXG there was an overall IOP change of - 4.8 mmHg (95% CI: - 7.5 to - 2.0).^[29]^

In most studies, patients with PEXG have a significantly greater drop in IOP after phacoemulsification than patients with POAG. Reduction of IOP has been previously reported in patients with PEXG after phacoemulsification was performed. It is suggested that phacoemulsification may remove the fibrillar material source in PEX eyes and facilitate aqueous humor drainage in the trabecular meshwork.^[25]^ Moreover, wider angles resulting from phacoemulsification and cataract removal increases the aqueous outflow from the TM and Schlemm canal.^[30]^ Therefore, in patients with significant cataract and uncontrolled glaucoma, performing phacoviscocanalostomy can result in better outcomes than performing VCS alone.

Comparable to previous reports, microperforation of the TDM was the most common intraoperative complication while fibrin formation was the most common postoperative complication of phacoviscocanalostomy.^[14,23]^

As compared to prior studies, the main advantage of our study in comparing phacoviscocanalostomy in PEXG and POAG was the use of a relatively higher number of cases with longer follow-up periods. According to literature, longer follow-up periods for patients tends to show a decrease in the success rate.^[12,22]^ We, however, demonstrated rather stable success rates after the 12 month follow-up session until the last follow-up visit at 40 months post operation.

Our study had several limitations. Firstly, IOP monitoring and grouping were done by an examiner who was not masked to the groups which might contribute to possible bias in the results. Considering the average follow-up time of this study, we recommend longer-term follow-up sessions for glaucoma patients undergoing phacoviscocanalostomy. It is also recommended that for better comparison of VCS in PEXG and POAG, the procedure should be performed in patients who have not had cataract removal.

Multiple factors may affect the reported success rate of phacoviscocanalostomy, including: preoperative IOP levels, different levels of the surgeons' experience, the surgical techniques chosen, the follow-up time, the various criteria used to determine the success rate and the patients' medical history. Longer follow-up durations could affect the success rate, mainly due to the time taken for the deposition of fibrillar material in the angle of pseudoexfoliative eyes and the scarring ostia of the Schlemm's canal.

Various definitions of the success rate, study designs, and the execution of multiple follow-up times are contributory factors toward making comparison between studies challenging.

For future studies, it is recommended that authors continue to utilize the international criteria for intraocular pressure (IOP) levels, to ensure that the comparison between studies remain practical.

In summary, phacoviscocanalostomy is a safe and effective procedure in achieving IOP reduction for both PEXG and POAG eyes, as it increases BCVA and decreases trabeculectomy-related complications. Therefore, this technique is recommended for eyes with therapeutically uncontrolled PEXG and POAG with cataract.

##  Financial Support and Sponsorship

None.

##  Conflicts of Interest

All authors declare that they have no conflicts of interests.
